# The Mechanics of Mercury in Syphilis

**Published:** 1903-03

**Authors:** Thomas W. Murrell

**Affiliations:** Richmond, Va., Lecturer on Dermatology and Assistant to the Chair of Genito-urinary Diseases, University College of Medicine


### THE MECHANICS OF MERCURY IN SYPHILIS.*

BY THOMAS W. MURRELL, M.D., RICHMOND, VA.,
Lecturer on Dermatology and Assistant to the Chair of Genito-urinary Diseases, University College of Medicine.

Mercury as used in the treatment of syphilis is one of the most ancient remedies. It was first used by the Chinese as a therapeutic measure in syphilis, and down through the ages up to this time it is the only drug that has answered the requirements as a means of treatment of this very ancient disease. Indeed mercury has been classified as the one of the two known specifics, and he is either a charlatan or an ignoramus who would dispute its preeminence in the therapy of syphilis. But, though this be admitted, there is a wide difference in the methods of treatment and the choice of the form of the drug to be used, and as to the last of these two, viz.: the form of the drug to be used, in this paper we will try and see by logical conclusions which is the best form to administer and our reasons for doing so.

First, let us look at the pathology of the disease. As in most things truly great it is exceedingly simple and consists solely of "an active proliferation of the normal germinal cells of the parts." When the virus of syphilis is introduced into the body there is on its development a stimulating effect produced on all the cells of the economy, some parts being affected more than others, and they begin to proliferate with exceeding rapidity. The lymph glands are attacked first and there is the familiar symptom of their general enlargement. As the source of inoculation is usually about the genitals the inguinal glands are the ones, of course, usually first affected, but if the inoculation is elsewhere the glands nearest the site of entry of the virus are the first to show enlargement. Then after the poison gets into the lymph channels it travels onward through the thoracic duct and into the subclavian vein where it is absorbed and finally affects all the tissues of the body. Yet, let it be thoroughly understood, there is no inflammation in the

* Read before the Richmond Academy of Medicine and Surgery January 27, 1903.

pathology of syphilis and the enlargement of the glands presents no sign of inflammation, the swelling being due to the increased number of cells in the gland and not to the swelling of the cells alone. This explains why the glands are not so much enlarged as they are hard and dense, and why the induration may remain after the initial lesion, the chancre, has disappeared; also, the same pathology applies to the eruption, which is not due to the excretion of the poison through the glands of the skin, as is the common belief among the laity, but is due to the proliferation of the epithelial cells, and this produces the shotlike feel that the eruption possesses in so many cases.

The lymphatics are affected and, the proliferating cells blocking up their channels, their function as sewers of the body is abolished, but the proliferation goes on elsewhere in the body, and the masses of cells not being carried off make their appearance as gumma in the tertiary stage. This is a result of syphilis and not a manifestation of the disease *per se*.

Now that we have looked into the pathology of this condition, I lay down a dictum somewhat surprising to the ordinary practitioner, to wit: Syphilis is as much a self-limited disease as measles, and that mercury has no antitoxic action upon the syphilitic virus. What then is its action? How are the results produced? Solely by mechanical means. We have seen that by the proliferation of these cells in the lymph channels that there is a blocking up of these sewers, these garbage carts of the body. Potassium iodide has a stimulating effect upon them, and since they are already stimulated to the limit by the virus of syphilis, this drug, as a therapeutic measure in the active stage of the disease, not only does no good but is capable of harm; you would be simply adding fuel to the fire. What you do desire is the potency of these lymph channels, something that will so keep them open that their function may go on uninterrupted.

If a gutter-pipe extending from the roof of this house to the ground were clogged up with filth so that the rain-water could not pass through, and you took a leaden ball about the size of the pipe and dropped it in it, by its own weight it would clean out the pipe and by opening up the channel restore its function. So in syphilis the globules of mercury in their passage through the lymph channels carry out the detritus before them and the function of the channel is preserved. If mercury is given in the crude form by mouth it passes through the intestinal tract unchanged, but if subdivided it is absorbed and has its effect; the finer the subdivision the quicker is its action made manifest and the more thorough the results. This is frequently practiced when in prescribing calomel you direct that it be triturated with white sugar, the fine subdivision of the calomel being the principle you are acting upon.

As a concrete drug mercury reflects all light and appears as silver to the eye; divided finer it appears white; finer still it takes on a grayish tinge, and fine as it can be divided it reflects all light and appears black to the eye. Give the crude drug, and, as we said, no results are produced; but give a coarse trituration to one person and a fine trituration to another for some length of time. If under autopsy a microscopical examination is made many more globules will be found in the glandular tissues of the person who took the fine subdivision than in the one to whom the coarse preparation was given; the drug always appearing as globules under the microscope, crowding each other through the finest channels. This teaches that the finely divided forms of the drug are the ones to use, because more is absorbed and quantity of absorption is the end to be attained.

The forms of mercury most used are the protoiodide or the biniodide, the bichloride, calomel, blue ointment and hydrarg. cum creta or mercury with chalk. We will take them up separately.

The protoiodide is the most used and at once the most useless. It is given in the mistaken idea that iodine does good, whereas we have seen that by stimulating the cells that are already overstimulated by the virus of syphilis that in the active stage it does harm. Dr. Louis Julien, one of the French authorities, in the *Journal de Maladies Cutanées et Syphilitiques* published lately, characterized the protoiodide as the "father of stomatitis," and I have seen in a clinic an iodine eruption in a person who was taking the protoiodide under a mistaken diagnosis of syphilis that simulated the syphilitic eruption in appearance and symmetry. Its action is uncertain, and as it was once a fad among the leading syphilographers, so now a reaetion has set in and it is beginning to be looked upon with suspicion.

At present the bichloride is being highly extolled for the injection treatment, but per os it has not proved satisfactory, being too severe a form of the drug. The blue ointment from its fine subdivision when used by inunction is perhaps the best, but the inunction treatment is not always feasible or desirable. This leaves us to the mild chloride and the mercury with chalk. Of these two forms more mercury can be given than in any other form by the mouth, and we have seen that quantity of absorption is the goal toward which we are striving.

In giving these the usual tonic treatment is carried out ; increasing the dose, to impending salivation and then cutting down to one-half for the regular dose to be taken from two to three years. At the end of this time, syphilis being worn out and dead, potassium iodide is indicated to stimulate the lymphatics and carry away the results of the disease, as the lymph channels you have kept open can be physiologically active.

It is unfortunate, I think, that mercury in any form does some good, because thereby we are deceived, but careful clinical observation has proved that in its milder forms the drug finely subdivided gives the best results.

BIBLIOGRAPHY.

*The Mercurials.* S. V. Clevenger.
*Journal American Medical Association,* Feb. 22 and 29, 1896.
White and Martin.
G. Frank Lydston.

*621 East Franklin Street.*

